# Evaluation of Uncertainties of the Null-Balanced Total-Power Radiometer System NCS1

**DOI:** 10.6028/jres.099.007

**Published:** 1994

**Authors:** Sunchana P. Pucic

**Affiliations:** National Institute of Standards and Technology, Boulder, CO 80303-3328

**Keywords:** calibration, coverage factor noise temperature, null-balanced, systematic effects, random effects, thermal noise, total power radiometer, uncertainty analysis

## Abstract

Standard uncertainties are evaluated for the null-balanced, total-power, heterodyned radiometer system with a switched input that was recently developed at NIST to calibrate thermal noise sources. Eight significant sources of uncertainty due to systematic effects are identified, two attributable to the two noise standards, and one each to connectors, the input mismatch, the input switch asymmetry, the isolator, the broadband mismatch and the attenuator. The combined standard uncertainty of a typical coaxial noise source calibration at a representative frequency of 2 GHz is about 1%, A strategy for reducing uncertainties is discussed.

## 1. Introduction

This article presents a detailed analysis of uncertainties associated with a new instrument (Noise Calibration System 1, or NCS1) for calibrating noise temperature. The instrument was recently developed at the National Institute of Standards and Technology, and is described in the accompanying articles [1] and [2]. Briefly, it consists of two noise standards and a total-power, null-balanced, heterodyned radiometer. One of the noise standards is a blackbody radiator at ambient temperature. In the present implementation of the system, the other standard is also a blackbody radiator, at liquid nitrogen temperature – the NIST primary coaxial cryogenic standard [3] However, any stable, calibrated noise source with noise temperature other than ambient and equipped with a proper connector can be substituted (with some degradation in performance, as discussed in this article).

A single-pole, triple-throw switch at the radiometer input provides dedicated ports for the two standards and the noise source under test (the sources). As presently implemented, NCS1 can calibrate coaxial noise sources with noise temperatures between cryogenic and 3 × 10^5^ K, in a frequency range from 1 GHz to 12 GHz. Access to an external vector network analyzer to characterize the system’s input ports is required.

A system equation describing the NCS1’s functioning [2] is amended to explicitly show the inherent uncertainty in the determination of the noise temperature of the unknown noise source:

$$T_{x}=T_{a}+\frac{M_{s}}{M_{x}}\cdot \frac{\eta {\mathrm{sw}}_{s}}{\eta {\mathrm{sw}}_{s}}\left(T_{s}-T_{a}\right)\frac{10^{-\left(A_{a}-A_{x}\right)/10}-1}{10^{-\left(A_{a}-A_{x}\right)/10}-1}\pm v_{c},$$ (1)

where

**Fig. 1:**
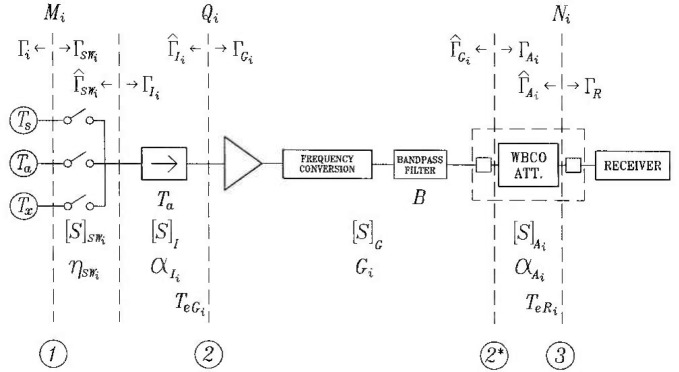
A block diagram of NCS1.

- *T_x_*, *T_a_* and *T_s_* are the available noise temperatures of the unknown noise source, the ambient temperature standard, and the nonambient temperature standard, respectively;
- *M_s_/M_x_* is a ratio of mismatch factors at the two input ports of the system dedicated to the non-ambient standard and the noise source under test (DUT). The two mismatch factors are defined as
  $$M_{i}=\frac{\left(1-{\left|\Gamma_{i}\right|}^{2})(1-|\Gamma_{sw_{j}}{|}^{2}\right)}{|1-\Gamma_{i}\Gamma_{sw_{j}}{|}^{2}},i=s,x,$$ (2)
  where *Γ*_i_ and ${\Gamma_{\mathrm{sw}}}_{j}$ are the reflection coefficients in Fig. 1.
- The term *η*sw*_s_*/*η*sw*_x_* is a ratio of the efficiencies of the two (slightly different) paths of the input switch, associated lengths of coaxial lines, and input connectors.
  An efficiency *η* is defined as a ratio of the power delivered to the output and the power delivered to the input of a linear two-port, $\eta =P_{{\text{del}}_{\text{out}}}/P_{{\text{del}}_{\text{in}}}$. Specifically, in the case of the input switch, assuming the isolator to provide infinite isolation,
  $${\eta_{\mathrm{sw}}}_{i}=\frac{\left(1-{\left|\Gamma_{i}\right|}^{2}\right)\cdot {\left|S_{21_{{\mathrm{sw}}_{j}}}\right|}^{2}}{\left(1-{\left|{{\hat{\Gamma }}_{\mathrm{sw}}}_{j}\right|}^{2}\right){\left|1-S_{11_{{\mathrm{sw}}_{j}}}\Gamma_{i}\right|}^{2}},i=s,x,$$ (3)
  where $S_{11_{{\mathrm{sw}}_{j}}}$ and $S_{21_{{\mathrm{sw}}_{j}}}$ are the *S*-parameters of the two paths of the input switch;
- The terms *A*_s_ and *A_x_* in Eq. (1) stand for the attenuator dial settings, in dB, needed to balance the system; and
- The last term explicitly denotes the combined standard uncertainty *v_c_* associated with the NCS1. It is addressed in the rest of the article.

The system equation [2] can be written in a shorthand form as

$$T_{x}=T_{a}+M\eta_{\mathrm{sw}}\left(T_{s}-T_{a}\right)Y.$$ (4)

The terms in Eq. (4) correspond to their counterparts in Eq. (1) in a straightforward manner; the uncertainty *v_c_* is omitted in Eq. (4). Both Eq. (1) without *v_c_* and Eq. (4) are referred to as “the system equation” and used in the rest of this article.

### 1.1 Method of Evaluating Uncertainties

Uniform distributions are assumed for the Type B evaluation of uncertainties [4] associated with systematic effects. The standard uncertainty is $a/\sqrt{3}$, where *a* stands for the mean value of the upper and lower limits of the distribution of the quantity in question. Small random variations of uncertain origin are assumed to be normally distributed, Their effects are quantified by the experimental standard deviation of the mean of *N* measurements [5], [6], which is Type A evaluation of uncertainty. Due to practical considerations and because the Type A uncertainty is small compared to the Type B uncertainties, a decision has been made to ordinarily make only five measurements.

In accordance with NIST policies [7], both the combined standard uncertainty *v*_c_ and the expanded uncertainty *U* are reported. The combined standard uncertainty *v*_c_ is calculated by the RSS method of combining Type A and Type B uncertainty components. The coverage factor *k* in the expanded uncertainty has the value of 2.

## 2. Standard Uncertainties Arising from Systematic Effects (Type B)

There are eight significant Type B uncertainties in the NCS1. Uncertainties that are much smaller than these eight are not discussed, with the exception of the nonlinearity uncertainty. This uncertainty is addressed because the assumption of the radiometer linearity is central to the derivation of the system equation.

In general, the system Eq. (1) (or its compact version, Eq. (4) is differentiated with respect to those input quantities that are explicitly present in the equation (the temperatures of the nonambient and ambient standards, the mismatch factor ratio, the input switch efficiency ratio, and the attenuation), In three instances the system equation needs to be first *corrected* for the input quantity in question (in the case of the uncertainties associated with the isolator, the connector, and the “broadband mismatch”), and then the uncertainty in the correction factor is evaluated.

### 2.1 Numerical Values

The calculation of the uncertainties, as performed by the NCS1 software, substitutes the actual values of those parameters that are measured, or the conservative estimates of those that are impractical to measure. In particular, complex reflection coefficients are measured and used in the calculations. However, to obtain numerical values for the individual uncertainties in the following sections, all values are chosen to be conservatively representative of parameters encountered during a typical measurement at the arbitrarily chosen frequency of 2.0 GHz. Since the phases of the reflection coefficients are too variable to justify using some typical value, magnitudes only are used in the calculations of the uncertainties involving reflection coefficients.

Calculations are performed for two different system configurations: when the nonambient noise standard is the primary NIST cryogenic coaxial standard, and when a commercial hot source is used instead. The cryogenic standard has a smaller uncertainty (at 2 GHz, *T*_s_= 83 K±2.9 K), but is not well matched (assumed |*T*_s_| = 0.2); the uncertainty of a commercial solid state noise source is necessarily larger (assumed to be *T_x_*=8400 K ± 140 K), but its impedance is fairly close to 50 *Ω* (assumed |*Γ_x_*| = 0.1).

The values of other input quantities entering into the calculations of most of the Type B uncertainties are: the temperature of the ambient standard *T*_a_=296 K, the magnitudes of the reflection coefficients of the radiometer port dedicated to the standard, $\left|\Gamma_{{\mathrm{sw}}_{s}}\right|=0.1$, and to the DUT, $\left|\Gamma_{{\mathrm{sw}}_{s}}\right|=0.1$.

Values of those input quantities that are particular to a specific uncertainty analysis are given in a corresponding section.

### 2.2 Nonambient Temperature Noise Standard

#### 2.2.1 NIST Primary Coaxial Cryogenic Noise Standard

If the NCS1 is configured with the NIST primary coaxial cryogenic noise standard serving as a nonambient standard, the uncertainty analysis proceeds as follows.

##### Source of Uncertainty

The uncertainty in the output temperature of the primary coaxial cryogenic standard, in kelvins, are given by [3]

$$\delta T_{s}=\pm 1.66\pm 0.85\sqrt{f},$$ (5)

where *f* stands for frequency in GHz.

##### Relative Standard Uncertainty

Assuming a uniform distribution, the relative standard uncertainty in the measurand $T_{x_{1}}$, in percent, due to the uncertainty in the temperature of the cryogenic standard *δT*, is given by

$$\frac{\delta T_{x}}{T_{x}}=\pm \frac{T_{s}}{T_{x}}\left|\frac{T_{x}-T_{a}}{T_{s}-T_{a}}\right|\frac{\delta T_{s}}{T_{s}}\cdot \frac{100\%}{\sqrt{3}}.$$ (6)

For typical values (Sec. 2.1), this standard uncertainty is 0.76%.

#### 2.2.2 Secondary Noise Standard

Any stable, calibrated noise source with noise temperature other than ambient and equipped with a proper connector can be used as a nonambient temperature standard in place of the NIST primary coaxial standard.

##### Source of Uncertainty

The source of uncertainty *δT*_s_, in kelvins, must be supplied by the calibration report accompanying the noise source which is to serve as the NCS1 standard. A typical commercial hot solid state noise source, calibrated at NIST at 2 GHz, may have its output reported as 8400 K ± 140 K.

##### Relative Standard Uncertainty

As in the case of the NIST cryogenic standard, the relative standard uncertainty, in percent, due to the uncertainty in temperature of the (secondary) noise standard, is given by

$$\frac{\delta T_{x}}{T_{x}}=\frac{T_{s}}{T_{x}}\left|\frac{T_{x}-T_{a}}{T_{s}-T_{a}}\right|\frac{\delta T_{s}}{T_{s}}\cdot \frac{100\%}{\sqrt{3}}.$$ (7)

For a typical value for the noise temperature of 8400 K ± 140 K, this standard uncertainty is 0.96%.

### 2.3 Ambient Temperature Standard

#### Source of Uncertainty

There are two sources of the uncertainty in the temperature of the ambient standard: the extent of a temperature gradient in the termination, and the measurement of the temperature.

The ambient temperature standard is contained in a temperature-controlled enclosure and isolated from potentially different temperatures at the radiometer input ports by a length of line and the input switch, both of which are contained in the same enclosure. Additionally, the temperature difference between the enclosure and the surrounding space is small, both temperatures being nominally ambient. Therefore, the temperature gradient in the ambient temperature standard is assumed to be negligible. Based on the type of the thermometer used to measure the ambient temperature, we can estimate this temperature with an uncertainty of *δT*_s_≈0.4 K.

#### Relative Standard Uncertainty

The relative standard uncertainty, expressed in percent, is

$$\frac{\delta T_{x}}{T_{x}}=\frac{T_{a}}{T_{x}}\left|\frac{T_{x}-T_{s}}{T_{a}-T_{s}}\right|\frac{\delta T_{a}}{T_{a}}\cdot \frac{100\%}{\sqrt{3}}.$$ (8)

For typical values from Sec, 2.1, this uncertainty is 0.11% for the NCS1 system equipped with the NIST primary cryogenic standard, and negligible if a commercial hot source is used instead.

### 2.4 Isolator

#### Corrected System Equation

The derivation of the system equation [Eq. (1)] is based on the assumption of perfect isolation of the radiometer input [2]. An isolator is inserted between the reference planes 1 and 2 (Fig. 1) to provide the desired isolation. However, the isolation of any isolator is noninfinite, and the resulting effects are discussed below.

In the following discussion, the input switch is assumed to be ideal (lossless and reflectionless) and therefore transparent, which results in the following simplifications: $\Gamma_{{\mathrm{sw}}_{i}}=\Gamma_{i_{j}}$, ${\bar{\Gamma }}_{{\mathrm{sw}}_{j}}=\Gamma_{i}$, and $\eta_{{\mathrm{sw}}_{i}}=1$. Furthermore, the heterodyning is assumed to be transparent, too.

The *corrected* system equation is derived following the procedure outlined in [2]. The two noise standards (with noise temperatures *T*_s_ and *T_a_*), and the unknown noise source (with noise temperature *T_x_*) are sequentially attached to the radiometer input, and the waveguide-below-cut-off (WBCO) attenuator is adjusted until the noise power delivered to the receiver *P*_dcl_ is the same in all three cases (*i* =s, a, *x*):

$$\begin{array}{l} \mathrm{kB}\{\left[T_{j}\alpha_{I_{j}}+T_{a}\left(1-\alpha_{I_{j}}\right)+T_{{\mathrm{eG}}_{j}}\right]G_{i}\alpha_{A_{j}}+\\ T_{a}\left(1-\alpha_{A_{j}}\right)+T_{{\mathrm{eR}}_{i}}\}N_{j}=P_{\text{del}}, \end{array}$$ (9)

where $\alpha_{I_{j}}$ and $\alpha_{A_{j}}$ are the available power ratios of the isolator and the attenuator, respectively, *G_i_* is the available gain of the section between the reference planes 2 and 2* (Fig. 1), $T_{{\mathrm{eG}}_{j}}$ and $T_{{\mathrm{eR}}_{j}}$ are the effective noise temperatures defined in the reference planes 2 and 3, and N_i_ is the mismatch factor in the reference plane 3.

Assuming, as in [2] that the attenuator is lossless $\left(\alpha_{A_{j}}=1\right)$, and that the system gain preceding it is high enough for the noise contribution of the receiver to be negligible $\left(T_{{\mathrm{eR}}_{j}}=0\right)$, the three equations become

$$\begin{array}{c} kB\left[T_{j}\alpha_{I_{j}}+T_{a}\left(1-\alpha_{I_{j}}\right)+T_{{\mathrm{eG}}_{i}}\right]G_{j}N_{j}=P_{\text{del}},\\ i=s,a,x \end{array}$$ (10)

The mismatch factor *N_j_* reduces to ${\left|S_{21_{A_{j}}}\right|}^{2}$ for a lossless attenuator inserted in the reflectionless environment [2]. Following the procedure described in [2] and neglecting third- and higher-order terms, we obtain the corrected system equation

$$\begin{array}{l} T_{x}=T_{a}+Y\frac{G_{s}}{G_{x}}\frac{\alpha_{I_{s}}}{\alpha_{I_{s}}}\left(T_{s}-T_{a}\right)+\frac{T_{a}}{G_{x}\alpha_{I_{x}}}\\ \left[Y\left(G_{s}-G_{a}\right)-\left(G_{x}-G_{a}\right)\right]+\\ +\frac{1}{G_{x}\alpha_{I_{x}}}\left[Y\left(G_{s}T_{{\mathrm{eG}}_{s}}-G_{a}T_{{\mathrm{eG}}_{a}}\right)-(G_{x}T_{{\mathrm{eG}}_{x}}-G_{a}T_{{\mathrm{eG}}_{a}})\right], \end{array}$$ (11)

where *Y* stands for

$$Y=\frac{\frac{{\left|S_{21_{A_{1}}}\right|}^{2}}{{\left|S_{21_{A_{1}}}\right|}^{2}}-1}{\frac{|S_{21_{A_{1}}}{|}^{2}}{|S_{21_{A_{1}}}{|}^{2}}-1}.$$ (12)

If the isolation were taken to be infinite, the available gain terms *G_j_* and the effective input noise temperatures $T_{{\mathrm{eG}}_{j}}$ would become independent of the input conditions, and the ratio $\alpha_{1_{x}}/\alpha_{1_{s}}$ would reduce to *M_x_*/*M_s_* [2]. Equation (11) would then become the system equation [Eq. (1)], adjusted to reflect the assumption of the ideal input switch (*η*s_s_/*η*sw_s_ = 1).

#### Source of Uncertainty

The correction (a full difference between the corrected and idealized system equations) consists of two terms:

$$\in_{l}=\in_{T_{\mathrm{eG}}}+\in_{G},$$ (13)

where $\in_{T_{\mathrm{eG}}}$ and *∈*_G_ describe the corrections due to the variability of the effective input noise temperature *T*_eG_ and the available gain *G*, respectively, as a function of the changes of the input impedance. Specifically

$$\begin{array}{l} \in_{T_{\mathrm{eG}}}=\frac{1}{G_{x}\alpha_{I_{s}}}[Y\left(G_{s}T_{{\mathrm{eG}}_{s}}-G_{a}T_{{\mathrm{eG}}_{s}}\right)-\\ \left(G_{x}T_{{\mathrm{eG}}_{s}}-G_{a}T_{{\mathrm{eG}}_{s}}\right)], \end{array}$$ (14)

and

$$\begin{array}{l} \in_{G}=\left(T_{x}-T_{a}\right)\left(\frac{G_{s}}{G_{x}}-1\right)\\ +\frac{T_{a}}{\alpha_{I_{s}}}\left[Y\left(\frac{G_{s}}{G_{x}}-\frac{G_{s}}{G_{x}}\right)-\left(1-\frac{G_{s}}{G_{x}}\right)\right] \end{array}$$ (15)

#### “Variable T_eG_” Correction

Experimental evaluation of the system has shown that changes of the effective input noise temperature with the maximal possible variation of the input impedance (a sliding short) is still below the system resolution; $\epsilon_{T_{\mathrm{eG}}}$ is therefore neglected, (that is, Eq. (14) becomes equal to zero), and Eq. (13) reduces to *ϵ*_I_ = *ϵ*_g_.

#### Variable G Uncertainly

The source of the uncertainty is the sensitivity of the available gain *G_i_*, in Eq. (15) to the changes in the input impedances *Γ_i_.* The available gain of the amplifier/left matching pad combination is given by

$$G_{j}=\frac{\left(1-{\left|{\bar{\Gamma }}_{I_{j}}\right|}^{2}\right){\left|S_{21_{G}}\right|}^{2}}{\left(1-{\left|{\bar{\Gamma }}_{G_{j}}\right|}^{2}\right){\left|1-S_{11_{G}}{\bar{\Gamma }}_{I_{j}}\right|}^{2}}$$ (16)

If third- and higher-order terms are neglected and the assumption that ${\bar{\Gamma }}_{G}\approx 0$ (assuring the reflectionless environment for the attenuator [2D is taking into account, the ratio of the available gain terms *G_s_/G_x_* in Eq. (15) becomes

GsGx≈1−|Γ¯Is|2+|Γ¯Is|2+2Re{(Γ¯Is−Γ¯Is)S11G}≈1+|S21S12|12(|Γx|2−|Γs|2)++2Re{(S21S12)1(Γs−Γx)(S22I*−S11G)}. (17)

An analogous expression holds for the *G_s_/G_x_* ratio in Eq. (15).

The term $|S_{12}S_{21}{{|}_{1}}^{2}$ in Eq. (17) is much smaller than unity even for a poor isolator, and, furthermore, it also multiplies a *difference* of the squares of the magnitudes of the reflection coefficients, so their product can be safely neglected. With that in mind, the worst-case expression for the *∈*_G_ term becomes

$$\begin{array}{l} \in_{G}\leq 2\left|T_{x}-T_{s}\right|{\left|S_{21}S_{12}\right|}_{I}\left(\left|S_{22_{I}}\right|+\left|S_{11_{a}}\right|\right)\left(\left|\Gamma_{s}\right|+\left|\Gamma_{x}\right|\right)+\\ +2\frac{T_{a}}{\alpha_{1_{x}}}{\left|S_{21}S_{12}\right|}_{I}\left(\left|S_{22_{I}}\right|+\left|S_{11_{G}}\right|\right)[Y\left(\left|\Gamma_{s}\right|+\left|\Gamma_{R}\right|\right)\\ +\left|\Gamma_{x}\right|+\left|\Gamma_{a}\right|]. \end{array}$$ (18)

#### Relative Standard Uncertainty

The quantity *ϵ*_G_ in Eq. (18) is taken to be uniformly distributed, with the relative standard uncertainty of

$$\begin{array}{l} \frac{\delta T_{x}}{T_{x}}=2\{\left|1-\frac{T_{a}}{T_{x}}\right|{\left|S_{21}S_{12}\right|}_{I}\left(\left|S_{22_{I}}\right|+\left|S_{11_{G}}\right|\right)\left(\left|\Gamma_{s}\right|+\left|\Gamma_{x}\right|\right)\\ +\frac{T_{a}}{T_{x}\alpha_{1_{x}}}{\left|S_{21}S_{12}\right|}_{I}\left(\left|S_{22_{I}}\right|+\left|S_{11_{G}}\right|\right)[Y(\left|\Gamma_{s}\right|\\ +\left|\Gamma_{s}\right|)+\left|\Gamma_{x}\right|+\left|\Gamma_{a}\right|\}\cdot \frac{100\%}{\sqrt{3}}. \end{array}$$ (19)

The factor *Y* is ordinarily measured. However, in order to estimate a typical uncertainty, it is here approximated [2] by

$$Y\approx \left|\frac{T_{x}-T_{a}}{T_{s}-T_{a}}\right|.$$ (20)

Under the following assumptions: an isolator with |*S*_21_ *S*_12_|_I_ ≈ 0.003 (corresponding to 50 dB isolation) and $\left|S_{22_{I}}\right|\approx 0.1$, $\alpha_{1_{s}}\approx 0.95$, |*Γ*_s_|≈0.01, $\left|S_{11_{G}}\right|\approx 0.1$, other values from the list of typical values in Sec. 2.1, the relative standard uncertainty is 0.05% for the NCS1 system equipped with the N1ST primary cryogenic standard, and 0.03% if a commercial hot source is used instead,

### 2.5 Connector

#### Corrected System Equation

Both the nonambient standard and the DUT have connectors that are reflective and lossy, those parameters are also partially nonrepeatable. The connectors are modeled as two-ports of efficiencies $\eta_{{\mathrm{CNN}}_{s}}$, and $\eta_{{\mathrm{CNN}}_{s}}$ that are each in cascade with the input ports of the radiometer. The corrected system equation becomes

$$T_{x}=T_{x}+\frac{\eta_{{\mathrm{CNN}}_{s}}}{\eta_{{\mathrm{CNN}}_{x}}}\cdot \frac{M_{s}}{M_{x}}\cdot \frac{\eta_{{\mathrm{sw}}_{s}}}{\eta_{{\mathrm{sw}}_{x}}}\left(T_{s}-T_{s}\right)Y.$$ (21)

#### Source of Uncertainty

Following the reasoning in [8], we assume that, for type *N* connectors, the efficiency ratio $\eta_{{\mathrm{CNN}}_{s}}/\eta_{{\mathrm{CNN}}_{x}}$ in Eq. (21) reduces to $\left(1+C_{1}\sqrt{f}+C_{2}\sqrt{f}\right)$, where the constants *C*_1_ and *C*_2_ have been experimentally determined to have the values of $C_{1}=0.00087/\sqrt{3}\}$ and *C*_2_=0.035, and *f* is the frequency in GHz. (For a connector with a center conductor different in diameter, the constant C_1_ is scaled by a factor (*D/D*_7mm_)^2^ since the loss in an uniform coaxial structure is proportional to the square of the magnetic field’s magnitude.)

The constant *C*_1_ encompasses components of connector variability arising from both systematic and random effects. Since the noise temperature measurements are performed without connecting and disconnecting the connectors (of the nonambient standard or the DUT), the random component cannot be assumed to average out.

The reference plane defined for calculating the connector uncertainty during a noise temperature measurement is assumed to be *to the left of the connector* in Fig. 1. In other words, a DUT is calibrated “stripped” of its own connector. In practice, the connector contribution to the combined standard uncertainty in noise measurements is so small for precision connectors in good condition and for frequencies in the microwave region of the spectrum that this assumption is admissible. The uncertainty arising in the connectors is at present an incompletely explored topic needing further study. The importance of resolving this issue becomes even greater with smaller connectors and higher frequencies.

#### Relative Standard Uncertainty

The entire correction (a difference between Eq. (21) and the system equation [Eq. (4)]) is uncertain, with *∈*_CNN_ having a uniform distribution with upper and lower limits of

$$\in_{\mathrm{CNN}}=\left(T_{x}-T_{s}\right)\left(1-\frac{\eta_{{\mathrm{CNN}}_{s}}}{\eta_{{\mathrm{CNN}}_{x}}}\right).$$ (22)

Substitution of experimental constants for the (I-efficiency ratio) term in Eq. (22) results in the relative connector standard uncertainty (in percent) of

$$\frac{\delta T_{x}}{T_{x}}=\left(\frac{0.087}{\sqrt{3}}+0.035\right)\sqrt{f}\left|1-\frac{T_{a}}{T_{x}}\right|/\sqrt{3}.$$ (23)

For typical values listed in Sec. 2.1, this standard uncertainty is 0.07% whether the NIST primary cryogenic standard or a commercial hot source is used.

### 2.6 Mismatch Factor

#### Source of Uncertainty

The mismatch factors between the nonambient standard and the radiometer, and between the DUT and the radiometer, are calculated from the reflection coefficient measurements [2]. Since only a *ratio* of the mismatch factors is used in the system equation, the uncertainties associated with the measurements of the reflection coefficients partially cancel.

The ratio of the mismatch factors *M_s_* and *M_x_* can be approximated in a well-matched system (|Γ|⪡1) by

$$\begin{array}{c} \frac{M_{s}}{M_{x}}≐1-{\left(a_{s}-a_{r_{s}}\right)}^{2}-{\left(b_{s}-b_{r_{x}}\right)}^{2}+\\ {\left(a_{x}-a_{r_{s}}\right)}^{2}+{\left(b_{x}+b_{r_{x}}\right)}^{2}, \end{array}$$ (24)

where *a_i_* and *b_i_* stand for the real and imaginary parts of the measured (complex) reflection coefficients: *Γ_s_* and *Γ_x_*, looking into the sources (the nonambient standard or the DUT), and $\Gamma_{{\mathrm{sw}}_{s}}$ and $\Gamma_{{\mathrm{sw}}_{x}}$, looking into the radiometer ports dedicated to the nonambient standard and the DUT. The relative uncertainty of that ratio can be approximated by

$$\frac{\delta \left(M_{s}/M_{x}\right)}{\left(M_{s}/M_{x}\right)}≐2\left(A+B\right),$$ (25)

where *A* and *B* are defined as

$$\begin{array}{l} A=\left(a_{x}-a_{{\mathrm{sw}}_{s}}\right)\left(\delta a_{x}-\delta a_{{\mathrm{sw}}_{s}}\right)-\\ \left(a_{s}-a_{{\mathrm{sw}}_{s}}\right)\left(\delta a_{s}-\delta a_{{\mathrm{sw}}_{s}}\right), \end{array}$$ (26)

$$B=\left(b_{x}+b_{{\mathrm{sw}}_{x}}\right)\left(\delta b_{x}+\delta b_{{\mathrm{sw}}_{x}}\right)-\left(b_{s}+b_{{\mathrm{sw}}_{s}}\right)\left(\delta b_{s}+\delta b_{{\mathrm{sw}}_{s}}\right).$$ (27)

It is assumed that the real and imaginary parts of the reflection coefficient are measured with an equal uncertainty of 0.005, which is independent of the magnitude of the reflection coefficients, as long as they are reasonably small (≤0.1 or so). With these provisions, Eq. (25) reduces to

$$\begin{array}{l} \frac{\delta M_{s}/M_{x}}{M_{s}/M_{x}}≐2\left|\left(b_{x}+b_{{\mathrm{sw}}_{x}}\right)-\left(b_{s}+b_{{\mathrm{sw}}_{s}}\right)\right|\\ \left(0.005+0.005\right). \end{array}$$ (28)

#### Relative Standard Uncertainty

The relative standard uncertainty in *T_x_* due to the uncertainty in the measurement of the reflection coefficients used in the calculation of the mismatch factor ratio, in percent, is given by

$$\frac{\delta T_{x}}{T_{x}}=\left|1-\frac{T_{s}}{T_{x}}\right|\frac{\frac{\delta M_{s}}{M_{x}}}{\frac{M_{s}}{M_{x}}}\cdot \frac{100\%}{\sqrt{3}}.$$ (29)

For typical values given in Sec, 2.1, this standard uncertainty is 0.07% for the NCS1 system equipped with the NIST primary cryogenic standard and 0.01% if a commercial hot source is used instead.

### 2.7 Asymmetry

#### Source of Uncertainty

The nonambient standard and the DUT are each attached to their own, dedicated input port. The input signals originating in the two sources, therefore, take slightly different paths to the rest of the radiometer. The two paths consist of a connector, a length of coaxial line, and one side of the input switch, and are modeled as two two-ports in cascade with the rest of the radiometer input [2]. The efficiencies of the two-ports, labeled “switch efficiencies” for short, enter into the system equation [Eq. (4)] as the *asymmetry correction η*sw*_s_*/*η*sw*_x_.*

The efficiency of a two-port depends on both th two-port and its environment, and so cannot b simply and directly measured. However, in a well matched system, the efficiency can be approx mated by

$$\eta *=\frac{{\left|S_{21}\right|}^{2}}{1-{\left|S_{11}\right|}^{2}},$$ (30)

which depends on the two-port alone and can be calculated from the *S*-parameters measured b: conventional methods.

It is convenient to express the ratio of the approximate efficiencies in logarithmic terms:

$$\frac{\eta_{sw_{s}}}{\eta_{sw_{x}}}=\frac{\eta_{sw_{s}}^{*}}{\eta_{sw_{x}}^{*}}=\frac{10^{\frac{\eta_{sw_{s}}^{*}}{10}}}{10^{\frac{\eta_{sw_{x}}^{*}}{10}}}=10^{\frac{\Delta \eta_{\mathrm{sw}}^{*}}{10}},$$ (31)

where $\Delta \eta_{S}^{*}w$ stands for the difference (in dB) of the approximate efficiencies of the two paths.

Equation (4) becomes

$$T_{x}=T_{a}+\frac{M_{s}}{M_{x}}\cdot 10^{\frac{\Delta \eta_{\mathrm{sw}}^{*}}{10}}\left(T_{s}-T_{a}\right)Y$$ (32)

Based on experimental data, the uncertainty in the measurements of $\Delta \eta_{\mathrm{sw}}^{*}$, $\delta \left(\Delta \eta_{\mathrm{sw}}^{*}\right)$, is estimated to be typically 0.01 dB for low-loss two-ports.

#### Relative Standard Uncertainty

The relative uncertainty in *T_s_* (in percent) due to the uncertainty in the measurement of the input switch asymmetry is given by

$$\frac{\delta T_{x}}{T_{s}}=\left|1-\frac{T_{a}}{T_{x}}\right|\frac{\ln10}{10}\cdot \delta \left(\Delta \eta_{\mathrm{sw}}^{*}\right)\cdot \frac{100\%}{\sqrt{3}}.$$ (33)

For typical values (Sec. 2.1), this standard uncertainty is 0.13% whether the NIST primary cryogenic standard or a commercial ‘hot source is used.

### 2.8 Broadband Mismatch

#### Corrected System Equation

A radiometer is a broadband instrument and ought to be described by a system equation that reflects that fact:

$$\int_{B}T_{x}gdf=\int_{B}\left[T_{a}+M_{\eta }Y\left(T_{s}-T_{a}\right)\right]gdf,$$ (34)

where *B* stands for the limiting system bandwidth, *M* is a ratio of mismatch factors at the reference plane 1 (Fig. 1), *η* is a ratio of the input switch efficiencies, and *Y* is the system balancing factor defined in Eqs. (4) and (1), The transfer function *g* is taken to be 1 within the band, and to vanish outside of the band.

The above equation reduces to Eq. (4) if all terms are independent of frequency. The noise temperatures *T_l_* are broadband by definition. Components of a noise measurement system determining the factor *Y* are manufactured to be broadband within the bandwidth of interest *B.* In the case of the NCS1, the mixer that downconverts the RF noise to the IF frequency, the WBCO attenuator used to balance the IF noise signals, and the square law detector that produces a dc output all satisfy the requirement. If the *η* term in Eq. (34) is also assumed to be broadband, the *corrected* system equation becomes

$$T_{x}=T_{a}+\frac{\int_{B}M_{s}gdf}{\int_{B}M_{x}gdf}\eta \left(T_{s}-T_{a}\right)Y.$$ (35)

The mismatch factors *M_s_*, and *M_x_* are not directly measured. Instead, the mismatch factors $M_{s_{0}}$ and $M_{x_{0}}$ are calculated from the measurements of the reflection coefficients, performed at the single (operating) frequency *f*_0_ Since reflection coefficients in general vary strongly with frequency, and the integration process indicated by Eq. (35) is not performed, an error issues.

#### Source of Uncertainty

The difference between the corrected and idealized system equations is taken to represent the limits of the broad band mismatch uncertainty; the quantity *∈*_bbm_ is assumed to be uniformly distributed, with upper and lower bounds of

$$\epsilon_{\mathrm{bbm}}=\left(T_{x}-T_{a}\right)\frac{\int_{B}M_{s}gdf/M_{s_{0}}}{\int_{B}M_{x}gdf/M_{x_{0}}}-1).$$ (36)

The mismatch factors $M_{i_{0}}$, *i*=s,*x* are a function of the reflection coefficients of the noise sources *Γ_i_* and of the radiometer input ports $\Gamma_{{\mathrm{sw}}_{j}}$ [2]. If the following assumptions can be made: an ideal (reflectionless, lossless, symmetrical) input switch, an infinitely isolating isolator, and a lossless uniform transmission line of a length *l* between the switch input and the isolator, the mismatch factors at the operating frequency *f*_0_ are given by

$$M_{0_{f}}=\frac{\left(1-{\left|\Gamma_{i}\right|}^{2}\right)\left(1-{\left|S_{11_{1}}\right|}^{2}\right)}{{\left|1-\Gamma_{i}S_{11_{1}}e^{-j^{\frac{4\pi d}{v}}f_{0}}\right|}^{2}},$$ (37)

where *ν* is the velocity of light in the transmission line.

**Fig. 2:**
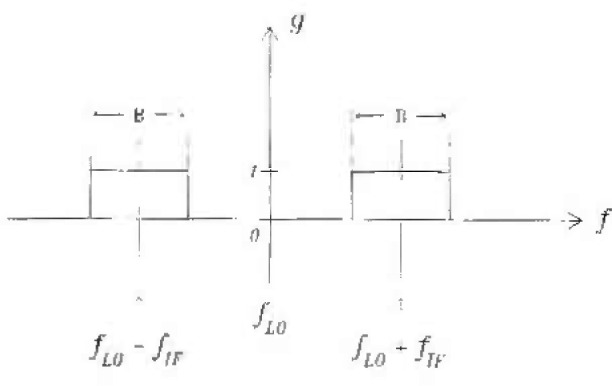
The integration range.

Substituting the Eq. (37) into Eq. (36), neglecting third- and higher-order terms, and performing the integration over the range in Fig. 2, we obtain the expression for the limits of broadband mismatch uncertainty [9].

∈bbm≤2|Tx−Ta|Re{S11t(Γs−Γx)}[1−cos(4mlvfIF)sinc(2πlvB)]. (38)

#### Relative Standard Uncertainty

The relative standard uncertainty due to broadband mismatch in a reasonably matched coaxial systems *δT_x_*/*T_x_*, in percent, is given by

δTxTx=2|1−TaTx|Re{S111(Γs−Γx)}[1−cos(4π1fIFv)sinc(2πlvB)]100%3. (39)

For the purpose of getting the numerical value here, the most unfavorable phasing of the reflection coefficients *Γ*_s_ and *Γ_x_* is assumed, resulting in the addition of their magnitudes. For the radiometer with a bandwidth of 4 MHz, an IF frequency of 30 MHz, and an isolator with the magnitude of its *S*_11_ parameter of 0.1, and a length of the line at the input of 0.2 m, the standard uncertainty is 0.1% with the NIST primary cryogenic standard, or 0.07% if a (better matched) commercial hot source is used.

### 2.9 Attenuator

The attenuator used in the NCS1, a 30 MHz, precision waveguide-below-cutoff attenuator, serves as a nulling device. The following analysis is valid when the attenuator is operated at an attenuation that is sufficiently high (above 20 dB, which, together with the insertion loss of 12 dB, puts the total *minimal* attenuation at 32 dB). At attenuation values lower than that, the deviation of the attenuation from linearity with respect to the coil separation distance is pronounced due to multiple modes being present.

The attenuator, with its tuned circuits at the input and output ports, has been observed to change its bandwidth as a function of the attenuation setting. A separate (system-limiting) 4 MHz bandpass filter, centered around 30 MHz has been therefore inserted ahead of the attenuator.

#### Source of Uncertainty

The uncertainty in the value of *A_i_* for a precision waveguide-below-cutoff attenuator is given in [10] by

$$\delta A_{i}=kA_{i}+C,$$ (40)

where *k* =0.0003, and *C* =0.003 dB.

#### Relative Standard Uncertainty

The relative standard uncertainty in the DUT’s noise temperature traceable to the attenuator is given by

$$\begin{array}{l} \frac{\delta T_{x}}{T_{x}}=\left|1-\frac{T_{a}}{T_{x}}\right|\frac{\ln10}{10}\cdot k[K_{I}\left(A_{x}-A_{a}\right)-\\ K_{2}\left(A_{s}-A_{a}\right)]\cdot \frac{100\%}{\sqrt{3}}, \end{array}$$ (41)

where

$$K_{I}=\frac{10^{-\left(A_{a}-A_{s}\right)/10}}{10^{-\left(A_{a}-A_{s}\right)/10}-1},$$ (42)

and

$$K_{2}=\frac{10^{-\left(A_{a}-A_{s}\right)/10}}{10^{-\left(A_{a}-A_{s}\right)/10}-1}.$$ (43)

Since only the differences in attenuation are used, the constant *C* in Eq. (40) drops out of consideration. For (experimentally obtained) values of attenuation of *A*_a_ = 22.87 dB, *A_x_* = 34.54 dB, and *A*_s_ = 20.95 dB, and with the typical values for other parameters from Sec. 2.1, the relative uncertainty is 0.06% for the NCS1 system equipped with the NIST primary cryogenic standard, and negligible if a commercial hot source is used instead.

### 2.10 Linearity

#### Source of Uncertainty

In order to accurately measure different noise power levels of the two noise standards and the DUT, a radiometer has to be linear within the specified dynamic range.

In the case of a balanced instrument such as the NCS1, the linearity requirement applies to the signal path up to and including the balancing mechanism. Referring to Fig. 1, the section of the NCS1 radiometer that needs to be linear extends between the input ports and the output of the WBCO attenuator. Any non linearity beyond the attenuator affects ail three noise powers equally (since they had been adjusted to the same level), and therefore cancels out.

The likely causes of deviation from linearity are: saturation in the RF amplifier; insufficient power of the local oscillator, causing the mixer to become nonlinear in the signal [11]; saturation in IF amplifiers preceding the attenuator; and a nonlinear behavior of the attenuator.

Instead of verifying the linearity of the individual components, the overall system linearity has been experimentally investigated. A fixed attenuator of approximately 3 dB is inserted between the isolator and the RF amplifier. A measurement of the noise temperature of the DUT is performed in the usual manner. The procedure is repeated a sufficient number of times and averaged to obtain a statistically convergent estimate of *T_x_*, The attenuator is then removed, and the measurements of *T_x_* repeated.

The obtained null difference has proven conclusively that no deviation from linearity exists, within the resolution of the NCS1 system.

## 3. Standard Uncertainties Arising from Random Effects (Type A)

The effects of the Type A uncertainties are quantified by calculating the standard deviation of the mean of *N* independent measurements of the noise temperature of the unknown source *T_x_* according to

$$\sqrt{\frac{\sum_{i=1}^{N}{\left(T_{x_{i}}-{\bar{T}}_{x}\right)}^{2}}{N\left(N-1\right)}}$$ (44)

where $T_{x_{i}}$ stands for the *i*th measured value and ${\bar{T}}_{x}$ for the mean of *N* measurements. The intermediate results of Eq. (44) are displayed after each measurement so the operator can select how many need to be performed. Since the Type A uncertainties are overshadowed by the Type B uncertainies in this system (by an order of magnitude), *N* can be chosen to be small (but no less than 5).

A representative value for the experimental standard deviation of the mean of five independent measurements of the noise temperature *T_x_* is 4 K for a 8400 K source, or 0.05 %.

## 4. Combined Uncertainty

The combined standard uncertainty is calculated by the RSS method. The coverage factor in the expanded uncertainty *k* is 2. Table 1 presents the typical uncertainties for a noise source having the noise temperature of 8400 K, measured at 2 GHz with either the NIST primary coaxial cryogenic standard, or a commercial hot source serving as a (non ambient) standard.

## 5. Discussion

Based on the results of the analysis of the uncertainties inherent to the measurement procedure, several comments regarding the performance of the NCS1 seem appropriate. The overall reported accuracy of the NCS1 is comparable to the accuracies of various noise measurement systems at NIST, if those were calculated by the same method. However, it compares unfavorably with the measurement accuracies of most other physical quantities. Some measures that would improve the NCS1 accuracy are discussed here.

From Table 1, the most effective measure to improve the overall accuracy of the NCS1 would be an improvement of the nonambient primary noise standard’s accuracy. The improvement persists whether a more accurate standard is used directly by NCS1 or a secondary commercial source, calibrated against a better primary standard having lower associated uncertainties, is used. A redesign of the primary noise standard is beyond the scope of this work, however.

Several standard uncertainties associated with the NCS1, as shown in Table 1, are proportional to the difference between the noise temperatures of the DUT and the nonambient standard. Relatively cool sources (such as commercially available hot/cold loads or solid state noise sources with *T_x_* = 1400 K) can be calibrated with better accuracy than more popular hot sources of *T_x_* ≈ 9000 K.

The NCS1 ambient standard accuracy can be improved readily, by using a better thermometer, by employing a four-wire resistance measuring technique, and by having an accurate voltmeter. A decrease in the uncertainty in the ambient standard from the present 0.4 K to 0.1 K would propagate into a decrease in the standard uncertainty arising from the ambient standard uncertainty from the present 0.11% to a negligible 0.03%.

Adding additional 25 dB of isolation practically eliminates the uncertainty traceable to isolation, but adds bulk and significantly increases the cost of the radiometer, since a separate extra isolator is needed for each octave.

The standard uncertainty associated with the broadband mismatch increases fast with the increasing length of the input transmission line, the IF frequency and the system bandwidth. The input section must be kept as compact as possible. A 5 MHz WBCO attenuator, replacing the 30 MHz attenuator used in the implemented NCS1, coupled with a 4 MHz bandpass filter, would reduce the standard uncertainty from 0.1% to a negligible 0.003%.

In some applications, overriding accuracy demands might prompt the elimination of the input switch. Although causing more connector wear and slowing the operations, it removes the uncertainty associated with switch asymmetry entirely and, by shortening the physical length of the input signal path, also decreases the uncertainty associated with the broadband mismatch. Additional small accuracy gain is made because the switch non-repeatability contribution to the Type A uncertainty is eliminated.

The uncertainty asssociated with random effects can be reduced in several ways. Selecting truly low-noise RF amplifiers with a sufficient gain (to make the mixer noise contribution negligible) would lessen the system noise contribution and therefore the random scatter. So would lowering the noise contribution of the passive components in the input section. Specifically, the switches need to be selected for their low losses, and the interconnecting coaxial lines kept as short as possible. An attempt to reduce the noise contribution by cooling the input section components must be done advisedly, however, since the derivation of the system equation presupposes them to be at the same temperature as the ambient standard.

The simple expedient of increasing the number of measurements also reduces the Type A uncertainty. Since each measurement compares the unknown noise temperature to that of the noise standards, the system drift over a longer period of time needed to perform an increased number of measurement is not a limiting factor. The gain in accuracy due to the random scatter reduction, marginal at present, might be worth the effort if the Type B uncertainties were reduced.

## Figures and Tables

**Table 1:** Typical NCS1 uncertainties with either a NIST primary coaxial standard or a calibrated hot noise source standard

Source of uncert.	NCS1 with NCST coax. standard	NCS1 with hot source standard
Nonambient std.	0.76%	0.96%
Ambient std.	0.11%	negl.
Isolator	0.05%	0.03%
Connector	0.07%	0.07%
Mismatch	0.07%	0.01%
Switch asymmetry	0.13%	0.13%
Broadband mismatch	0.10%	0.07%
Attenuator	0.06%	negl.
Linearity	negl.	negl.
Random effects	0.03%	0.03%

Comb. std. uncert.	0.80%	0.97%

Expanded Uncert.	1.60%	1.95%
